# Taxonomic study of the planthopper genus *Lacusa* Stål, 1862 (Hemiptera, Fulgoromorpha, Lophopidae)

**DOI:** 10.3897/zookeys.405.6981

**Published:** 2014-04-28

**Authors:** Jichun Xing, Xiangsheng Chen

**Affiliations:** 1 Institute of Entomology, Guizhou University, Guiyang, Guizhou, P. R. China, 550025; 2 College of Animal Sciences, Guizhou University, Guiyang, Guizhou, P. R. China, 550025; 3 Special Key Laboratory for Development and Utilization of Insect Resources of Guizhou, Guiyang, Guizhou, P. R. China, 550025

**Keywords:** Auchenorrhyncha, morphology, taxonomy, distribution, new species

## Abstract

The planthopper genus *Lacusa* Stål, 1862 is reviewed. All species are illustrated and male genital characters are provided, and including two new species: *L. digitata* **sp. n.** and *L. producta* **sp. n.** from Yunnan Province, China. The species *L. orientalis* Liang, 2000 is removed from this genus based on the frons with median carina and fore femur and tibia flattened but not foliaceous. A key to species is also given. The type specimens of the new species are deposited in the Institute of Entomology, Guizhou University, Guiyang, China (GUGC).

## Introduction

The planthopper genus *Lacusa* was established by Stål (1862) for a single species *Elasmoscelis fuscofasciata* Stål, 1854 from India. Later, Atkinson (1886) placed *Cixius eminens* Walker, 1858 as a junior synonym of *Lacusa fuscofasciata* (Stål, 1854). Chou & Huang (see Chou et al. 1985) described a new species *Lacusa yunnanensis* from China. Liang (1996) proposed *Lacusa yunnanensis* Chou & Huang, 1985 as a junior synonym of *Lacusa fuscofasciata* (Stål, 1854). Liang (2000) placed *Sarebasa* Distant, 1909 as a junior subjective synonym of *Lacusa*, 1862 and proposed the new combination *Lacusa celebris* (Distant, 1909), and described a new species *Lacusa orientalis* from Laos, Vietnam and China. Later, Soulier-Perkins (2001) reinstated *Sarebasa* as a separate genus based on the phylogenetic analysis in Lophopidae.

Here, we follow Soulier-Perkins (2001) in treating *Sarebasa* as a genus distinct from *Lacusa* based on the morphological characteristics for these two genera (see Table 1). The species *Lacusa orientalis* Liang, 2000 indicates that the original figures and description are not belong to the genus *Lacusa* based on the frons with median carina and fore femur and tibia flattened but not foliaceous, and it may be belong to the genera *Sarebasa* or *Acothrura*, and its placement is not treated further here. Consequently, this genus now contains only one species: *Lacusa fuscofasciata*.

**Table 1.** Differences among *Lacusa* with *Pitambara* and *Sarebasa*.

	*Lacusa*	*Pitambara*	*Sarebasa*
1. Body length (from apex of vertex to tip of forewings, male)	Less than 10 mm	Less than 10 mm	More than 10 mm
2. Length of vertex	Broader than long	Longer than broad	Broader than long
3. Midian carina on the frons	Absent	Present	Present even if rudimentary
4. Median anterior margin of pronotum	Regularly rounded	Anteriorly protuberant	Regularly rounded
5. Fore femur and tibia	Foliaceous	Flattened but not foliaceous	Relatively elongate, but not foliaceously dilated
6. Hind tibiae with lateral spines (number)	3	2	3

In this paper, two new species *Lacusa digitata* sp. n. and *Lacusa producta* sp. n. are described and illustrated from Yunnan Province, China. The type specimens of the new species and other materials examined are deposited in the Institute of Entomology, Guizhou University, Guiyang, China (GUGC). The genus *Lacusa* now contains 3 species, a key is given to separate all species.

## Material and methods

Specimens were collected by sweeping net. Dry specimens were used for the description and illustration. External morphology was observed under a stereoscopic microscope and characters were measured with an ocular micrometer. Color pictures for adult habitus were obtained by KEYENCE VHX-1000 system. The genital segments of the examined specimens were macerated in 10% NaOH and drawn from preparations in glycerin jelly using a Leica MZ 12.5 stereomicroscope. Illustrations were scanned with Canon CanoScan LiDE 200 and imported into Adobe Photoshop CS8 for labeling and plate composition. Morphological terminology follows Liang (2000).

## Taxonomy

### Lacusa

Stål

http://species-id.net/wiki/Lacusa

Lacusa Stål, 1862: 309; Atkinson 1886: 42; Distant 1906: 323; Muir 1930: 478; Chou et al. 1985: 125; Liang 2000: 283.

#### Type species.

*Elasmoscelis fuscofasciata* Stål, 1854

#### Description.

Body length (from apex of vertex to tip of forewings) less than 10 mm, size medium. Head short, approximately trapezoidal, ratio width of vertex from base to length in middle line 1.6, narrower than pronotum; vertex broader than long, anterior margin straight in dorsal view and not produced anteriorly beyond proximal margin of eyes, lateral margins with carinate, with an obsolete median longitudinal carina. Frons with lateral carinae, and with sublateral carinae fused apically, and without median carina. Rostrum extending to meso-trochanter, with ratio subapical to apical segment 2.0. Pronotum slightly longer than vertex, anterior margin roundly produced, posterior margin approximately straight, tricarinate on disc, lateral areas curved down. Mesonotum broad, with tricarinate on disc. Forewings broadly round at apex, outer and inner margins nearly parallel, precostal area with many oblique transverse parallel crossveins; hindwings narrower than forewings. Legs moderately long, fore femora and tibiae foliaceous. Hind tibiae with 3 lateral spines and 3–4 rows with more than 70 small spines apically, apical spines of first hind tarsal segment separated by a pad of microsetae.

Head pale luteous, suffused with piceous brown. Eyes black brown. Frons brown and anteclypeus piceous brown. Antennae black brown. Rostrum yellowish brown. Forewings semiopaque, with three broad fuscous transverse band and irregularly piceous spots; hindwings semihyaline. Fore and middle legs blackish brown; hind legs luteous, pad yellowish. Abdomen piceous brown.

*Male genitalia*. Pygofer short, upper 1/3 very narrow in lateral view, without appendage. Anal tube longer than pygofer, apex forked in caudal view. Genital styles short to long, dorsolateral with a small hook or a finger-like process near posterior margin. Aedeagus with 2 dorsally directed, spinose processes, dorsally directed at medioventral margin; near base of aedeagus on dorsal side or mediolateral of aedeagus with 2 spinose processes or not; base of aedeagus on dorsal side with 1 spinose process or not, and each base laterally of aedeagus with 1 processes or not.

#### Diagnosis.

The genus *Lacusa* resembles *Pitambara* Distant, 1906 and *Sarebasa* Distant, 1909 in having the vertex not produced anteriorly beyond proximal margin of eyes, apical spines of first hind tarsal segment separated by a pad of microsetae, and frontal disc not longitudinally deeply concave, but differs from these genera by the characters noted in Table 1.

#### Distribution.

China, India, Nepal, Burma, Thailand.

#### Key to species (male) of *Lacusa* from China

**Table:** 

1	Aedeagus stout, its base with 1 spinose process on dorsal side and base laterally without process	2
–	Aedeagus long and curved in the middle, its base without spinose process on dorsal side and each base laterally with 1 process	*Lacusa fuscofasciata*
2	Mediolateral of aedeagus with 2 long spinose processes	*Lacusa producta* sp. n.
–	Near base of aedeagus on dorsal side with 2 short spinose processes	*Lacusa digitata* sp. n.

### Lacusa fuscofasciata

(Stål, 1854)

http://species-id.net/wiki/Lacusa_fuscofasciata

Figs 1–10

Elasmoscelis fuscofasciata Stål, 1854: 248.
Cixius eminens Walker, 1858: 42 (synonymized by Atkinson 1886: 42).
Lacusa fuscofasciata (Stål, 1854), comb. n. by Stål, 1862: 309; Atkinson 1886: 42; Distant 1906: 324, Fig. 159; Melichar 1915: 355; Liang 2000: 287, Figs 10, 18–22.
Lacusa yunnanensis Chou & Huang (in Chou et al.) 1985: 128, 137-138, Fig. 119 (synonymized by Liang 1996: 147).

#### Material examined.

1♂, China: **Guizhou** Province, Jiangkou County, Fanjingshan National Natural Reserve, 24 September 2011, coll. Weibin Zheng (GUGC); 26♂♂14♀♀, China: **Guizhou** Province, Wangmo County, 19 August 2012, coll. Zhimin Chang, Weibin Zheng, Jiankun Long and Weicheng Yang (GUGC); 4♂♂1♀, China: **Guizhou** Province, Ceheng County, 25 August 2012, coll. Zhimin Chang, Weibin Zheng and Jiankun Long (GUGC); 2♂♂, China: **Guizhou** Province, Luodian County, 16 May 2013, coll. Jiankun Long (GUGC); 6♂♂6♀♀, China: **Hainan** Province, Jianfengling National Natural Reserve, 9 April 2013, coll. Jiankun Long, Jichun Xing and Yubo Zhang (GUGC); 1♂, China: **Yunnan** Province, Ruili City, Nongdao, 15 July 2013, coll. Haiyan Sun (GUGC).

#### Distribution.

China (Yunnan, Guizhou, Guangdong, Hainan), India, Nepal, Burma, Thailand.

#### Note.

Chou & Huang (in Chou et al. 1985) described a new species *Lacusa yunnanensis* from Menglun, Xishuangbanna, Yunnan Province, China, based on one female specimen, and they noted that *Lacusa yunnanensis* was closely related to *Lacusa fuscofasciata*, but that it could be distinguished from it by the fuscous transverse band near outer margin on forewing branched and the fuscous meso- and metathorax. Later, Liang (1996) proposed *Lacusa yunnanensis* as a junior synonym of *Lacusa fuscofasciata* based on examination of specimens from Yunnan, Guizhou, Guangdong and Hainan Provinces in China.

Body length (from apex of vertex to tip of forewings) of male specimen from Guizhou and Hainan Provinces, relatively small about 6.9 mm, but male specimen from Yunnan Province relatively larger about 8.0 mm.

**Figures 1–10.**
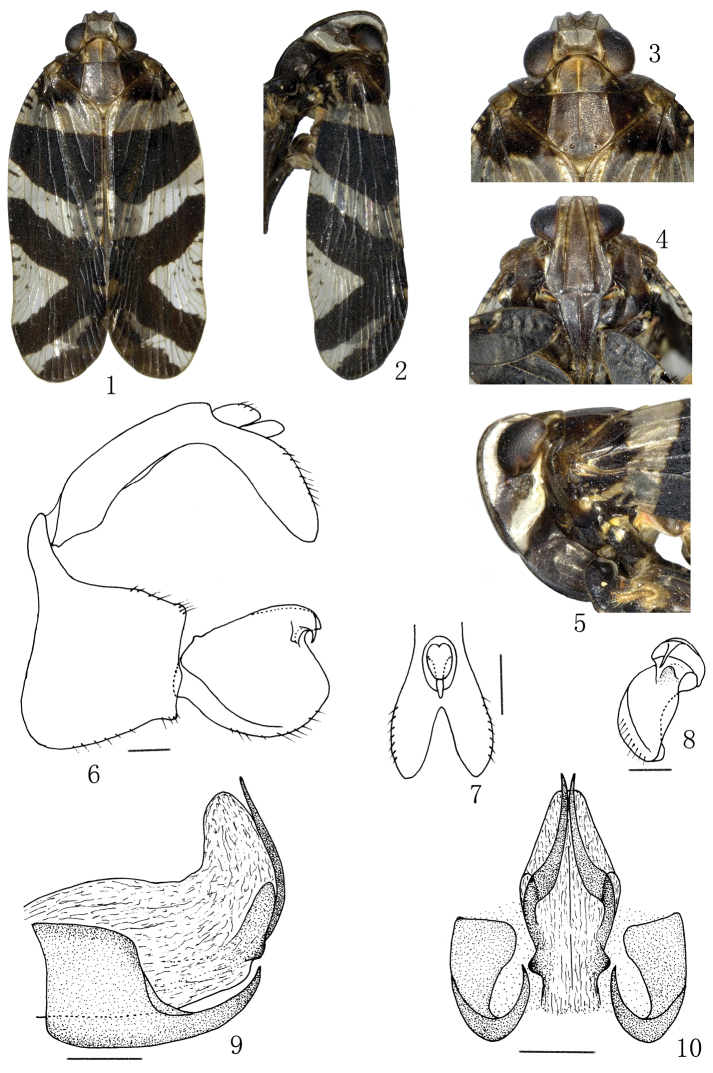
*Lacusa fuscofasciata* (Stål, 1854). **1** Male habitus, dorsal view **2** Male habitus, lateral view **3** Male, head and thorax, dorsal view **4** Male, frons and clypeus **5** Male, head and thorax, lateral view **6** Male genitalia, lateral view **7** Apex of anal tube, caudal view **8** Left genital style, caudal view **9** Aedeagus, lateral view **10** Aedeagus, caudal view. Scale bars: **6–10** = 0.20 mm.

### Lacusa digitata

Xing & Chen sp. n.

http://zoobank.org/9CAB07EA-9508-458D-836B-A312AEC90CA1

http://species-id.net/wiki/Lacusa_digitata

Figs 11–22

#### Description.

Body length (from apex of vertex to tip of forewings): male 8.5–8.7 mm (n=2).

**Figures 11–22.**
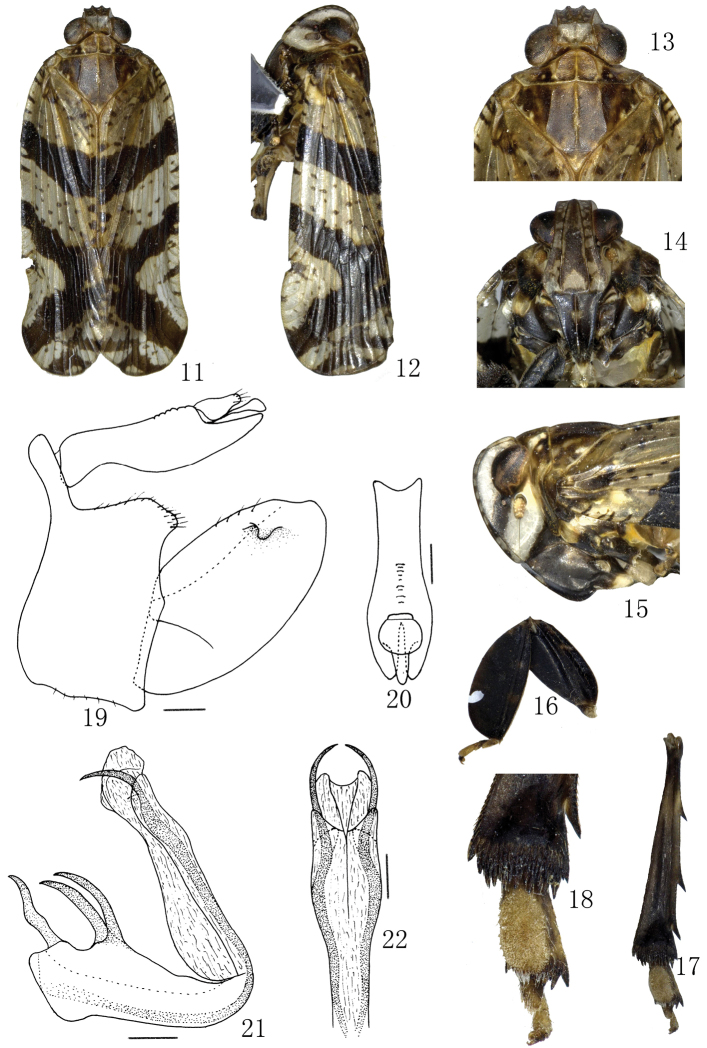
*Lacusa digitata* sp. n. **11** Male habitus, dorsal view **12** Male habitus, lateral view **13** Male, head and thorax, dorsal view **14** Male, frons and clypeus **15** Male, head and thorax, lateral view **16** Left fore femur and tibia, dorsal view **17** Left hind tibiae and lateral spines, and tarsomere, dorsal view **18** Apex of left hind tibiae and tarsomere, dorsal view **19** Male genitalia, lateral view **20** Anal tube, dorsal view **21** Aedeagus, lateral view **22** Aedeagus, caudal view. Scale bars: **19–22** = 0.20 mm.

Color pattern of anterior dorsum and face as in Figs 11–15. Pronotum and mesonotum yellowish brown. Tegula yellowish. Ocelli red. Apical margin of forewings maculately piceous. External features as in generic description.

*Male genitalia*. Pygofer short, upper 1/3 very narrow in lateral view, dorsal margin strongly concave (Fig. 19). Anal tube in dorsal view with ratio length to maximum width 3.0 (Fig. 20). Genital styles long, apex approximately round in lateral view, dorsolateral with a finger-like process near posterior margin (Fig. 19). Aedeagus with 2 dorsally directed, spinose processes, dorsally directed at medioventral margin, and exceed the end of aedeagal shaft; near base of aedeagus on dorsal side of aedeagus with 2 short spinose processes; base of aedeagus on dorsal side with 1 short spinose process (Figs 21, 22).

#### Type material.

Holotype: ♂, China: **Yunnan** Province, Ruili City, Mengla County, Moli, 5 June 2011, coll. Jiankun Long (GUGC); paratypes: 1♂, **Yunnan** Province, Lianghe County, Mengyang, 27 July 2013, coll. Zhihua Fan (GUGC).

#### Diagnosis.

This species is similar to *Lacusa fuscofasciata* (Stål, 1854) but can be distinguished by the base of aedeagus on dorsal side with one spinose process, near base of aedeagus on dorsal side with two short spinose processes, the anal tube relatively short, and the genital styles much more long.

#### Etymology.

The species name is derived from the Latin word “*digitata*”, indicating that the genital style dorsolateral with a finger-like process near posterior margin.

### Lacusa producta

Xing & Chen sp. n.

http://zoobank.org/D770CD3E-2AC6-4C93-9A7D-AF6AEE307EA4

http://species-id.net/wiki/Lacusa_producta

Figs 23–31

#### Description.

Body length (from apex of vertex to tip of forewings): male 9.2–9.3 mm (n=3).

**Figures 23–31.**
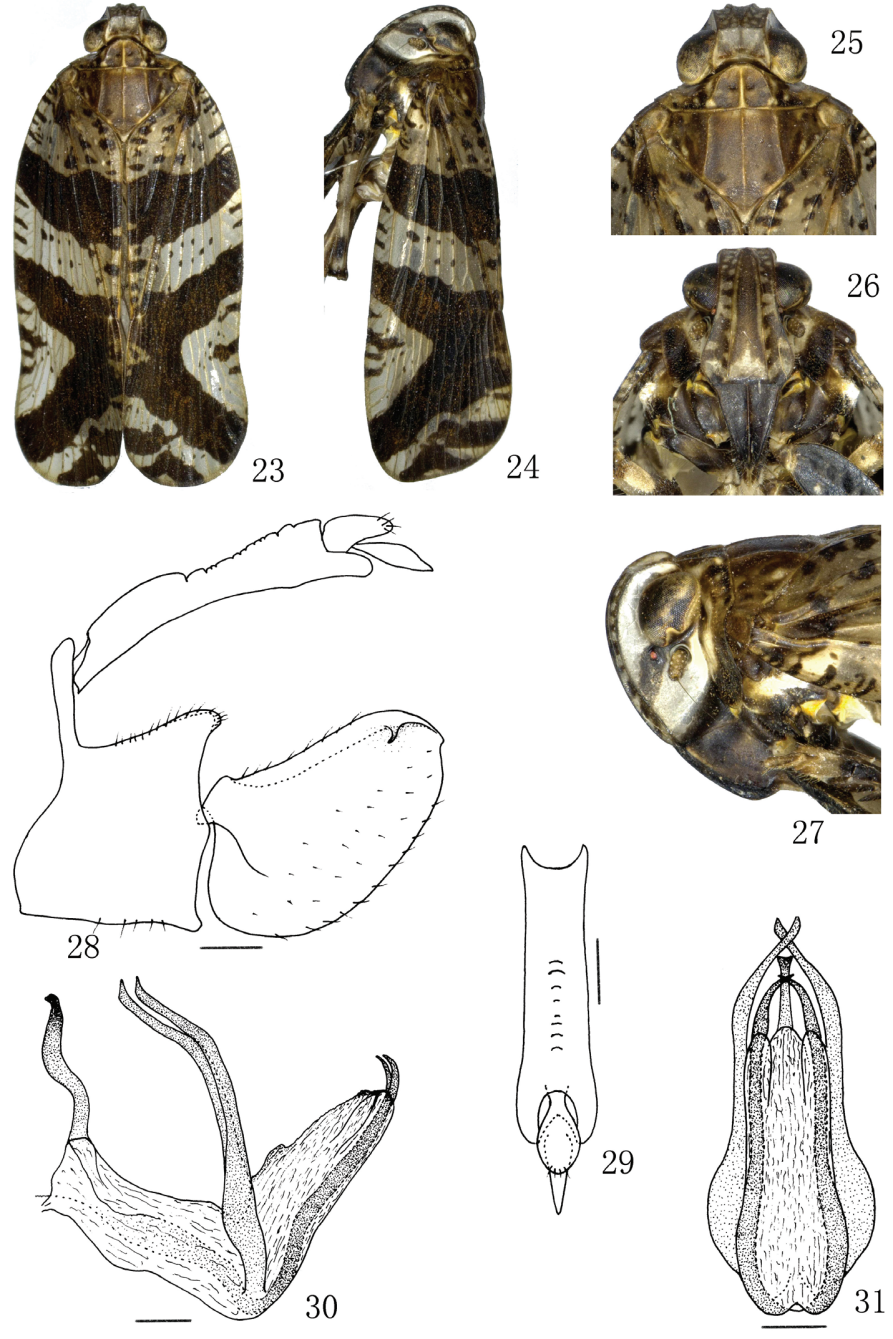
*Lacusa producta* sp. n. **23** Male habitus, dorsal view **24** Male habitus, lateral view **25** Male, head and thorax, dorsal view **26** Male, frons and clypeus **27** Male, head and thorax, lateral view **28** Male genitalia, lateral view **29** Anal tube, dorsal view **30** Aedeagus, lateral view **31** Aedeagus, caudal view. Scale bars: **28–31** = 0.20 mm.

Color pattern of anterior dorsum and face as in Figs 23–27. General appearance as in *Lacusa digitata* sp. n., but the body much larger.

*Male genitalia*. Pygofer short, upper 1/3 very narrow in lateral view, dorsal margin strongly concave, posterior margin angle producted near the middle; (Fig. 28). Anal tube in dorsal view with ratio length to maximum width 4.1 (Fig. 29). Genital styles long, apex approximately round in lateral view, dorsolateral with a small hook process near posterior margin (Fig. 28). Aedeagus with 2 dorsally directed, spinose processes, dorsally directed at medioventral margin, and exceed the end of aedeagal shaft; mediolateral of aedeagus with 2 long spinose processes; base of aedeagus on dorsal side with 1 spinose process (Figs 30, 31).

#### Type material.

Holotype: ♂, China: **Yunnan** Province, Xishuangbanna, Mengla County, Mohan, 25 July 2013, coll. Jichun Xing (GUGC); paratypes: 2♂♂, **Yunnan** Province, Xishuangbanna, Mengla County, Mohan, 25 July 2013, coll. Yuan Liu and Yangyang Liu (GUGC).

#### Diagnosis.

This species is similar to *Lacusa digitata* sp. n. but can be distinguished by the mediolateral of aedeagus with two long spinose processes, genital style dorsolaterally with a small hook process near posterior margin, the anal tube relatively long.

#### Etymology.

The species name is derived from the Latin word “*producta*”, indicating that the mediolateral of aedeagus with two long spinose processes.

## Supplementary Material

XML Treatment for Lacusa

XML Treatment for Lacusa fuscofasciata

XML Treatment for Lacusa digitata

XML Treatment for Lacusa producta

## References

[B1] Atkinson ET (1886) Notes on Indian Rhynchota. No. 5. Journal and Proceedings of the Asiatic Society of Bengal 55: 12-83

[B2] Chou I Lu JS Huang J Wang SZ (1985) Homoptera: Fulgoroidea. Science Press, Beijing, China, 152 pp. [In Chinese with English summary]

[B3] Distant WL (1906) The fauna of British India, including Ceylon and Burma. Rhynchota 3 (Heteroptera-Homoptera). Taylor & Francis, London, 503 pp.

[B4] Distant WL (1909) Rhynchota malayana Part II. Records of the Indian Museum 3: 163-181

[B5] Liang AP (1996) Taxonomic changes in Chinese Lophopidae with a check list of Chinese species (Homoptera: Fulgoroidea). Pan-Pacific Entomologist 72: 145-151

[B6] Liang AP (2000) Oriental Lophopidae: new taxa and taxonomic changes (Insecta: Hemiptera: Fulgoroidea). Reichenbachia 33: 281-311

[B7] Melichar L (1915) Monographie der Lophopinen. Annales Historico Naturales Mu- sei Nationalis Hungarici 13: 337-384

[B8] Muir FAG (1930) On the classification of the Fulgoroidea. Annals and Magazine of Natural History (Ser.10) 6: 461–478

[B9] Soulier-Perkins A (1998) The Lophopidae (Hemiptera: Fulgoromorpha): description of three new genera and key to the genera of the family. European Journal of Entomology 95: 599-618

[B10] Soulier-Perkins A (2001) The phylogeny of the Lophopidae and the impact of sexual selection and coevolutionary sexual conflict. Cladistics 17: 56-78. doi: 10.1111/j.1096-0031.2001.tb00111.x

[B11] Stål C (1854) Nya Hemiptera. Ofversigt af Kongliga Svenska Vetenskaps-Akademiens Förhandlingar 11: 231-255

[B12] Stål C (1862) Novae vel minus cognitae Homopterorum formae et species. Berliner Entomologische Zeitschrift 6: 303-315

